# Unraveling Neurodegeneration: Common Molecular Mechanisms and Novel Therapeutic Concepts in Major Neurodegenerative Disorders

**DOI:** 10.1002/brb3.71615

**Published:** 2026-08-25

**Authors:** Fatemeh sadat Aldaghi, Zakieh Siahpoosh, Zivar Salehi, Farhad Mashayekhi, Shabnam Sohrabnezhad

**Affiliations:** ^1^ Department of Biology, Faculty of Science University of Guilan Rasht Iran; ^2^ Faculty of Chemistry University of Guilan Rasht Iran

**Keywords:** disease‐modifying therapies, mitochondrial dysfunction, neurodegenerative diseases, neuroinflammation, oxidative stress, protein aggregation

## Abstract

**Purpose:**

Although Alzheimer's disease (AD), Parkinson's disease (PD), amyotrophic lateral sclerosis (ALS), multiple sclerosis (MS), and Huntington's disease (HD) present with markedly different clinical phenotypes, these neurodegenerative diseases (NDDs) appear to converge on a shared set of underlying molecular disturbances. This review sought to integrate disease‐specific causative triggers with shared pathogenic pathways, focusing on neuroinflammatory signaling, oxidative imbalance, mitochondrial impairment, and disrupted protein homeostasis, in order to support multi‐target, disease‐modifying therapeutic strategies.

**Method:**

Relevant classical and contemporary literature, encompassing original research and review articles on the molecular basis of AD, PD, ALS, MS, and HD, was reviewed and synthesized narratively, with attention to how neuroinflammatory and oxidative stress pathways intersect, reinforce one another through mitochondrial and inflammasome‐driven feedback, and recur across the five conditions.

**Finding:**

In each disorder, persistently activated microglia and astrocytes secreted inflammatory mediators and reactive oxygen species, engaged the NLRP3 inflammasome, and progressively destabilized cellular homeostasis through a self‐perpetuating cycle linking neuroinflammation and oxidative stress. Disease‐specific lesions nonetheless persisted: amyloid‐β and tau pathology in AD; α‐synuclein aggregation with iron‐driven mitochondrial damage in PD; RNA‐binding protein dysfunction, proteostatic collapse, and excitotoxicity in ALS; inflammatory demyelination and axonal bioenergetic failure in MS; and mutant huntingtin‐driven transcriptional and mitochondrial disruption in HD. These distinct triggers ultimately converged on shared downstream cascades.

**Conclusion:**

Recognizing this shared pathogenic foundation supports multi‐target therapies—such as Nrf2 activation, NLRP3 inhibition, mitochondria‐targeted antioxidants, and gene‐based interventions—that extend across diagnostic boundaries, though challenges in intervention timing, patient stratification, and clinical translation remain unresolved.

## Graphical abstract

Neurodegenerative diseases share common pathological mechanisms including chronic neuroinflammation, oxidative stress, mitochondrial dysfunction, and protein aggregation. Despite distinct hallmarks in Alzheimer's, Parkinson's, Huntington's, ALS, and MS, these processes converge, driving progressive neuronal damage and neurodegeneration.

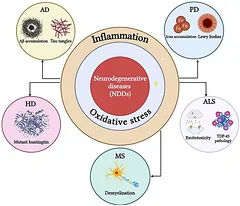

## 1 Introduction

Neurodegenerative diseases (NDDs) rank among the most significant medical and societal challenges today, impacting tens of millions of people globally. Current data show that more than 70 million individuals are affected worldwide, and these numbers have steadily increased over the last 30 years (Jiang et al. 2025). These disorders involve the gradual death of neurons in both the central nervous system (CNS) and peripheral nervous system (PNS). The main types include Alzheimer's disease (AD), Parkinson's disease (PD), Huntington's disease (HD), amyotrophic lateral sclerosis (ALS), multiple sclerosis (MS), and several others, such as frontotemporal dementia (FTD) and prion diseases (Denaro et al. 2025).

Although each NDD has unique clinical features, they share important underlying mechanisms, primarily protein misfolding and aggregation, mitochondrial impairment, oxidative stress, and sustained neuroinflammation (Antico et al. 2025; Tesco and Lomoio 2022).

**FIGURE 1:**
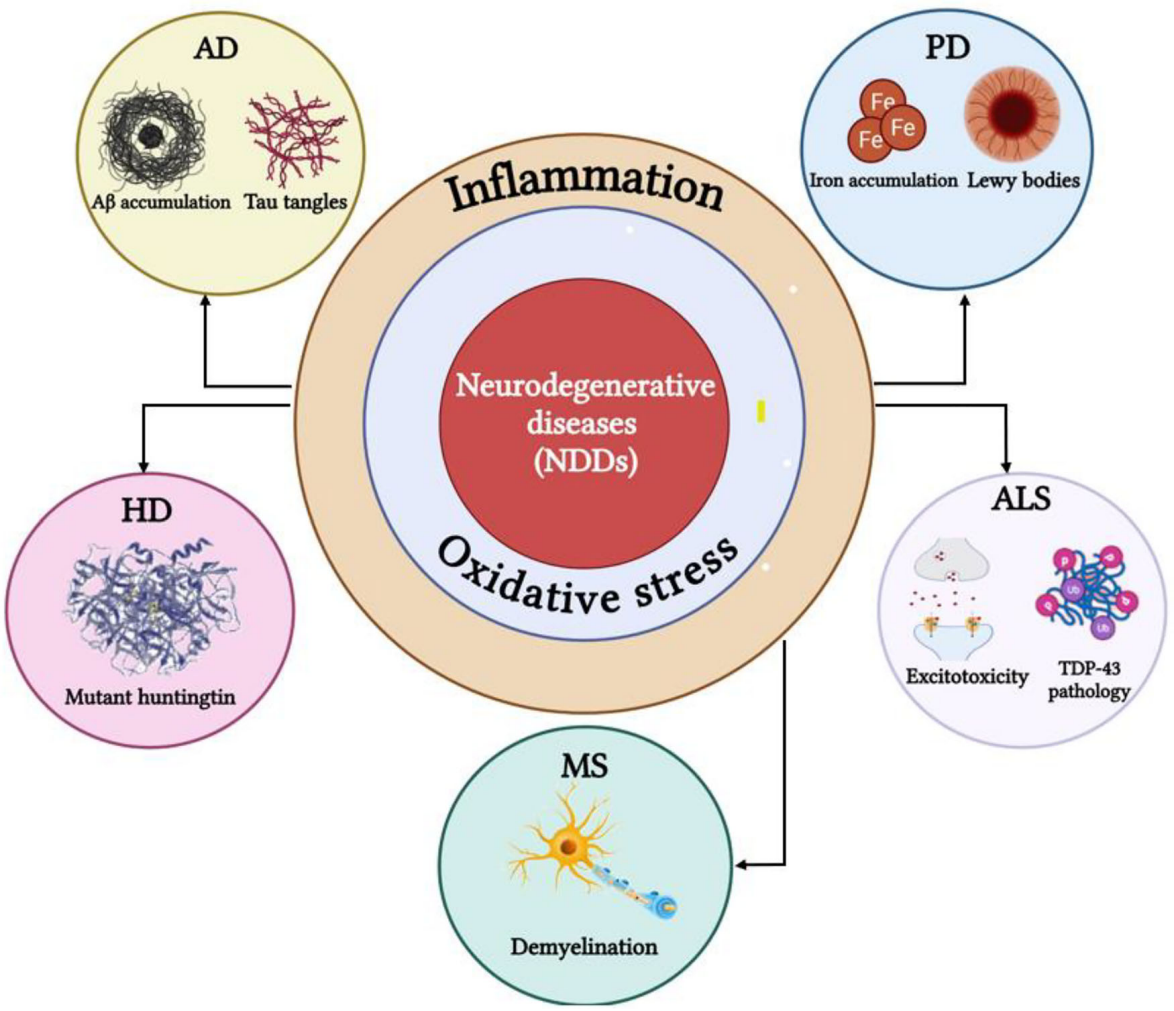
Comprehensive illustration of shared pathological mechanisms in neurodegenerative diseases (NDDs). NDDs exhibit several overlapping pathological processes, including chronic neuroinflammation, oxidative stress, mitochondrial dysfunction, impaired proteostasis, and protein aggregation. Although each disorder is uniquely defined by specific pathological hallmarks—such as amyloid‐β (Aβ) deposition and tau tangles in AD, iron accumulation and Lewy bodies in PD, mutant huntingtin aggregates in HD, TDP‐43 abnormalities and excitotoxicity in ALS, and demyelination in MS—these distinct features ultimately converge on shared mechanisms. The interplay between inflammation, oxidative stress, and mitochondrial failure drives progressive neuronal dysfunction and eventual neurodegeneration across these conditions.

Existing drugs mostly relieve symptoms temporarily but fail to slow or stop disease progression. This shortcoming has directed scientific efforts toward therapies that can modify the disease course by targeting these core molecular drivers (Chen et al. 2026) (Figure 1)

To advance our understanding of NDDs and support the development of effective treatments that reduce symptoms, it is essential to identify the factors that drive disease pathogenesis and the molecular mechanisms underlying them. A further challenge is to translate this knowledge into novel therapeutic strategies, while also clarifying how the immune system contributes to neurodegenerative processes and the loss of nervous system function.

## 2 Common Pathogenic Mechanisms in Neurodegenerative Diseases

### 2.1 Neuroinflammation: A Double‐Edged Sword in the Central Nervous System

Neuroinflammation refers to an immune response occurring within the CNS (encompassing the brain and spinal cord) primarily mediated by microglia and astrocytes, but also involving infiltrating peripheral immune cells and a broad array of inflammatory mediators (Almikhlafi 2026). Under physiological conditions, it contributes to homeostasis by promoting pathogen clearance, debris removal, and tissue repair. However, when persistent stimuli such as damage‐associated molecular patterns (DAMPs), protein aggregates, or misfolded proteins sustain this response, neuroinflammation becomes maladaptive and drives a self‐perpetuating cycle of glial activation, cytokine release, oxidative stress, and progressive neurodegeneration (Alves de Lima et al. 2020; Zang et al. 2022).

#### 2.1.1 Reactive Astrocytes and Cytokine Signaling

Astrocytes are star‐shaped cells that have a common origin with neurons (Tchieu et al. 2019). In the early stages of CNS development, these cells are derived from neural progenitor cells and differentiate into mature astrocytes during late embryonic and early postnatal development (Baldwin et al. 2024). In addition to immune function, astrocytes play other roles, including supporting the trophic system of neurons, regulating ion concentrations, balancing neurotransmitters, and BDNF secretion (Albini et al. 2023).

Astrocytes are part of the CNS and can release chemokines, cytokines, and other immune factors. In a resting state, astrocytes secrete factors such as granulocyte‐macrophage colony‐stimulating factor (GM‐CSF), granulocyte colony‐stimulating factor (G‐CSF), and TGF‐β (Choi et al. 2014). After neuronal injury, they are activated and respond to DAMPs or pathogen‐associated molecular patterns (PAMPs) through Toll‐like receptors (TLRs) (Bsibsi et al. 2002; Fischer et al. 2021). Astrocytes activated in response to neuroinflammation have shown significant functional heterogeneity that leads to exacerbation or improvement of neuronal injury (López‐Teros et al. 2024).

The response of these receptors activates intracellular signaling pathways, such as the transcription factors of the nuclear factor kappa B (NF‐κB) family, thereby facilitating inflammatory cytokine expression, such as tumor necrosis factor alpha (TNF‐α), IL‐1β, IL‐6, and chemokines such as CXCL8, CCL3, and CCL2 (Y. Zhao et al. 2024). In pathological states, reactive astrocytes critically modulate disease progression by secreting molecular signals that alter blood–brain barrier (BBB) permeability, call in immune cells from the blood, and shape inflammation inside the brain. Depending on the microenvironmental stimuli, these cells exhibit either neuroprotective or neurotoxic phenotypes; however, in chronic neurodegenerative contexts, the neurotoxic functional state predominantly prevails (Fan and Huo 2021).

#### 2.1.2 Microglial Activation and Cytokine Signaling

Microglia are resident macrophages in the CNS derived from mesoderm. Specifically, microglia originate from primitive macrophages in the yolk sac during embryonic development and seed the CNS before the BBB forms (Ginhoux et al. 2010; Utz et al. 2020). Microglia express a variety of membrane receptors for chemicals, including cytokines, chemokines, and neurotransmitters, enabling them to respond rapidly to disturbances in brain homeostasis (Paolicelli et al. 2022). In physiological conditions, these cells play an essential role in maintaining homeostasis, protecting against pathogenic agents, and repairing neuronal damage (Fleiss et al. 2021). Microglia serve context‐dependent roles in CNS homeostasis. In the healthy adult brain, they support neuronal plasticity and contribute to myelin maintenance by phagocytosing surplus oligodendrocytes and clearing damaged myelin (Ma et al. 2025; Rasia‐Filho et al. 2023). However, under pathological conditions, the microglial cells can shift toward neurotoxic activity, underscoring that their net effect on tissue integrity depends on the nature and persistence of the activating stimulus rather than a fixed functional identity (Ayata et al. 2025; Ennerfelt et al. 2022; Fang et al. 2023). Microglia continuously survey the CNS microenvironment and rapidly respond to changes in tissue homeostasis. Following injury or pathological stimulation, they migrate to the affected site and undergo functional changes that can support tissue repair or, under persistent activation, contribute to inflammatory damage. Rather than fitting into fixed M0, M1, and M2 categories, microglia display dynamic and context‐dependent states that shape disease progression through cytokine release, phagocytic activity, and interaction with neurons and other glial cells (Leyh et al. 2021).

### 2.2 Oxidative Stress and Redox Imbalance in Neurodegeneration

Helmut Sies first used the term “oxidative stress” to describe the phenomenon of an imbalance between the production and removal of reactive oxygen species (ROS) and reactive nitrogen species (RNS) in cells and tissues, which may contribute to the degeneration of biological systems (Forman and Zhang 2021; Sies and Cadenas 1985). ROS is produced in two main ways: endogenous and exogenous pathways (Bkaily et al. 2026). Mitochondrial electron transport chains (ETCs) and enzymatic reactions rank among the primary endogenous sources. Exogenous sources include ionizing radiation, ultraviolet radiation, and oxidizing chemicals (Kotha et al. 2022). At low concentrations, ROS acts as a secondary messenger in cellular signaling; however, prolonged exposure to high concentrations damages cellular macromolecules such as lipids, proteins, and DNA. This damage is therefore linked to the induction of irreversible cell death (necrosis) or to programmed pathways of cell death (apoptosis). Reactive species play a critical role in the oxidation of LDL, which facilitates the onset and progression of atherogenic damage and cardiovascular disease (Kotha et al. 2022).

Antioxidants are the cellular defense system against reactive species. These molecules play a protective role by neutralizing the cell's ROS and RNS. The antioxidants include glutathione peroxidase (GPx), superoxide dismutase (SOD), catalase (CAT), reduced glutathione (GSH), proteins like albumin and metallothionein, fat‐soluble vitamins like D, A, and E, and water‐soluble vitamins like C (Nolfi‐Donegan et al. 2020; Orywal et al. 2025).

*SOD*: A neuroprotective enzyme that recycles harmful O_2_^−^ to reduce the toxicity of hydrogen peroxide (H_2_O_2_). Three types of SOD have been identified: (1) SOD1, a soluble enzyme that is associated with mutations in ALS (Berdyński et al. 2022). (2) SOD2 (manganese‐containing type): This class is in the mitochondria, and dysfunction can lead to diseases such as cardiomyopathy, motor neuron disorders, and cancer. (3) SOD3: This class is an extracellular superoxide dismutase (EC‐SOD) and is known as the most abundant type of SOD. This class is present in peripheral tissues and is associated with heart and lung diseases (Chidambaram et al. 2024).

*CAT*: Catalase is one of the most critical antioxidant enzymes found in almost all aerobic organisms. This enzyme decomposes two H_2_O_2_ molecules to one O_2_ molecule and two H_2_O molecules in two steps, which contributes to reducing cytotoxicity (Glorieux and Calderon 2017).

*GPx*: Glutathione peroxidase is another crucial antioxidant enzyme that reduces hydrogen peroxide and organic hydroperoxides using GSH as an electron donor, playing a central role in protecting neurons from oxidative damage and maintaining cellular redox homeostasis (Averill‐Bates 2023).

Increased levels of oxidative stress have serious consequences for the biological system. Studies have shown that oxidative stress is associated with several diseases, including cancer, cardiovascular disease, hypertension, diabetes, NDDs, and aging (Anderson et al. 2018; S. Liu, He, et al. 2025). Cellular death is due to a number of mechanisms, including oxidative stress. Accumulation of ROS is a primary mechanism. Mitochondrial function is impaired due to this accumulation (N. Liu, Liu, et al. 2025). ROS causes toxicity, activates cytokines, interferes with iron metabolism, and induces cellular processes such as necroptosis and pyroptosis (Sikder et al. 2025). The accumulation of ROS in neurons causes high levels of intracellular glutamate and calcium (Ca^2+^) to be released, disrupting homeostasis and stimulating neuroinflammation. ROS production increases as a result of neuroinflammation, exacerbating cytotoxicity (Hroudová and Fišar 2025; Sanabria‐Castro et al. 2024). Rather than serving as benign metabolic byproducts, ROS and RNS actively propagate neurodegeneration via four primary pathogenic axes. First, they accelerate the aberrant aggregation of proteins like Aβ, tau, and α‐synuclein, which clump together (Hilt et al. 2018). Second, they damage the lipid membranes around synapses, which weakens signaling between neurons (Sun et al. 2024). Third, they disrupt the structural integrity of the axonal transport machinery (Wali et al. 2020). Fourth, distinct neuronal populations exhibit differential susceptibility compared to others; dopamine neurons in PD and motor neurons in ALS have weak antioxidant defenses and lots of iron, so they die first (Gonzalez‐Rodriguez et al. 2020).

### 2.3 Interplay of Neuroinflammation and Oxidative Stress

Neuroinflammation and oxidative stress share a bidirectional, self‐amplifying relationship in neurodegeneration (He et al. 2020). Microglial activation via TLRs or DAMPs leads to M1 polarization, upregulating NADPH oxidase 2 (NOX2) and iNOS, resulting in ROS and RNS bursts. Astrocytes also critically contribute: reactive astrocytes release IL‐6, TNF‐α, and ROS via NOX4‐dependent pathways, while impaired glutamate uptake (downregulation of EAAT1/EAAT2) leads to excitotoxicity, further amplifying neuronal ROS production (López‐Teros et al. 2024; Zhao et al. 2024).

Mitochondria serve as both sources and targets of this interplay. Damaged mitochondria release mitochondrial DNA (mtDNA) and cardiolipin (mitochondrial DAMPs), which activate the NLRP3 inflammasome within microglia. NLRP3 activation cleaves pro‐IL‐1β and pro‐IL‐18 into active cytokines, driving chronic neuroinflammation (Amo‐Aparicio et al. 2023). Conversely, pro‐inflammatory cytokines (TNF‐α, IL‐1β) impair mitochondrial complex I and IV activity, increasing electron leak and ROS generation, creating a feed‐forward loop (Fabisiak and Patel 2022).

**FIGURE 2:**
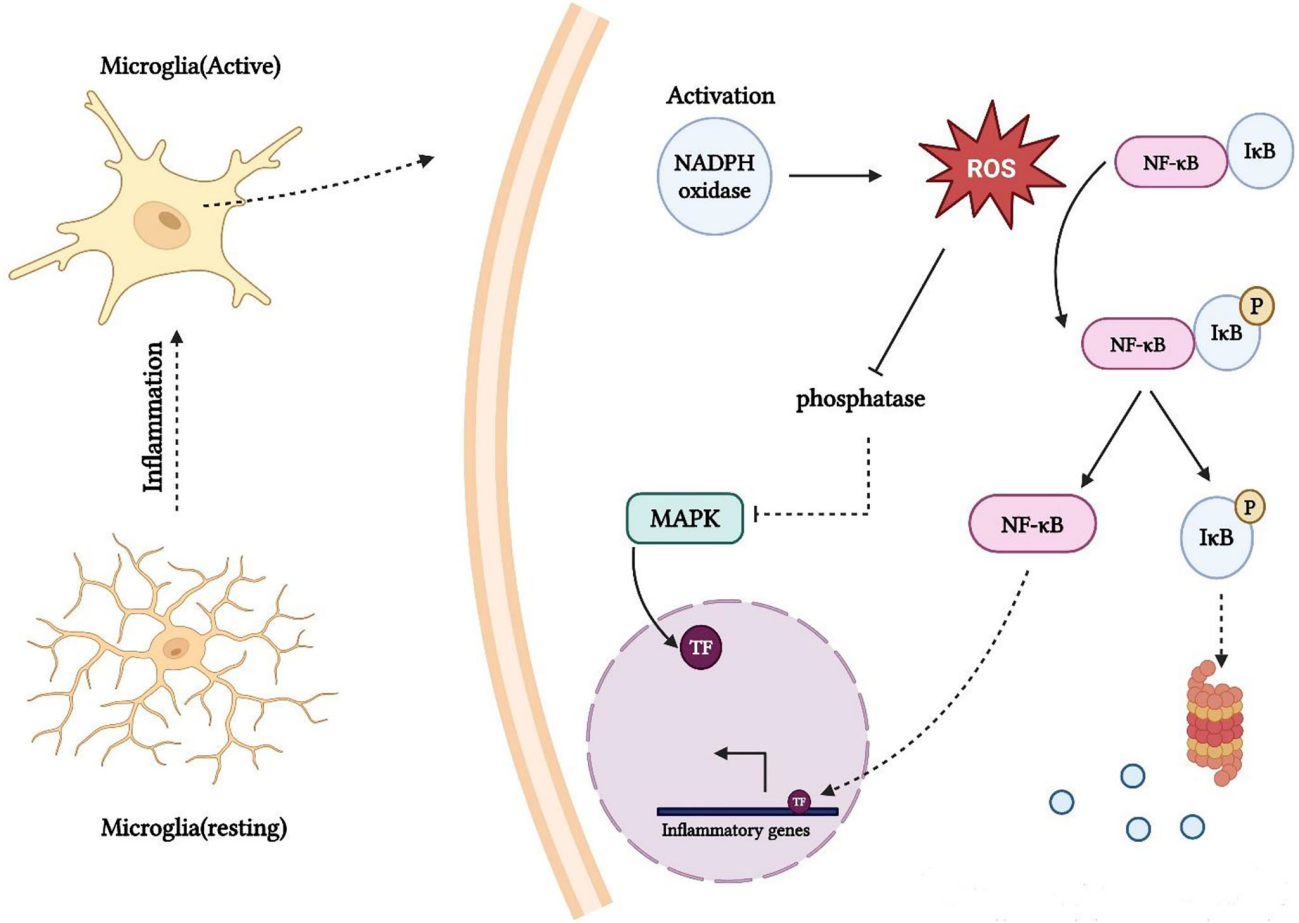
The relationship between oxidative stress and inflammation in the activation of microglia. The activation of NADPH oxidase in microglia leads to the generation of ROS, which serve as crucial signaling molecules linking oxidative stress to inflammatory responses. ROS inhibit phosphatase activity, thereby maintaining MAPK activation and promoting the transcription of inflammation‐related genes. Furthermore, ROS supports the phosphorylation and subsequent proteasomal degradation of IκB, allowing NF‐κB to translocate to the nucleus and enhance the production of pro‐inflammatory mediators. The concurrent activation of the MAPK and NF‐κB pathways exacerbates neuroinflammation, propelling microglia into a heightened state of activation.

Redox‐sensitive NF‐κB acts as a molecular switch: ROS activate IKK complex → IκB degradation → NF‐κB nuclear translocation → upregulation of IL‐1β, IL‐6, TNF‐α, COX‐2, iNOS, further amplifying both inflammation and oxidative stress. Thus, neuroinflammation and oxidative stress are not separate entities but two facets of a unified pathogenic cascade (Figure 2).

## 3 Pathogenic Factors in Specific Neurodegenerative Diseases

### 3.1 Alzheimer's Disease: Architects of Pathogenicity

#### 3.1.1 Disease‐Specific Initiating Events and Pathogenic Cascade

AD is the most prevalent neurodegenerative disorder worldwide, currently affecting over 55 million individuals, with projections estimating a rise to 139 million by 2050. This escalating prevalence imposes an immense socioeconomic and clinical burden globally. Pathologically, AD is characterized by extracellular amyloid‐β (Aβ) plaques and intracellular neurofibrillary tangles (NFTs) composed of hyperphosphorylated tau protein (Zhang et al. 2024).

The cascade initiates via the amyloidogenic processing of amyloid precursor protein (APP) by β‐secretase (BACE1) and the γ‐secretase complex, liberating hydrophobic Aβ42 peptides (Hardy and Higgins 1992; Vassar et al. 1999). Soluble Aβ oligomers, rather than insoluble plaques, exert the primary synaptotoxic effects by binding to N‐methyl‐D‐aspartate receptors (NMDARs) and cellular prion protein (PrP^C^) (Laurén et al. 2009). This interaction drives abnormal Ca^2+^ influx, which aberrantly activates downstream kinases, including glycogen synthase kinase‐3 beta (GSK3β) and cyclin‐dependent kinase 5 (CDK5) (Shankar et al. 2008). These kinases directly mediate the hyperphosphorylation of tau, causing its detachment from microtubules, which disrupts axonal transport and promotes its aggregation into NFTs (Iqbal et al. 2005). This pathological accumulation is accelerated by the APOE‐ε4 allele, which severely impairs LRP1‐mediated endocytic clearance of Aβ (Prasad and Rao 2018; Tachibana et al. 2019).

#### 3.1.2 Integration With Shared Neuroinflammatory Signaling

Extracellular Aβ oligomers act as DAMPs that activate microglial TLRs (TLR2/4) and CD36, stimulating the NF‐κB pathway to upregulate pro‐IL‐1β transcription (Stewart et al. 2010). Concurrently, microglial phagocytosis of Aβ induces lysosomal destabilization and cathepsin B leakage, which triggers the assembly of the NLRP3 inflammasome and caspase‐1 activation, liberating mature IL‐1β and IL‐18 (Heneka et al. 2013; Yuyama et al. 2024). Chronic secretion of IL‐1β and TNF‐α induces the polarization of neighboring astrocytes into the neurotoxic A1 phenotype, leading to a loss of homeostatic synaptic support (Liddelow et al. 2017). This neuroinflammatory milieu forms a feed‐forward loop; elevated TNF‐α upregulates BACE1 expression, while inflammatory signaling drives GSK3β activation, accelerating both Aβ production and tau hyperphosphorylation. Non‐steroidal anti‐inflammatory drugs repress BACE1 transcription via NF‐κB signaling (Sastre et al. 2006).

#### 3.1.3 Integration With Mitochondrial Failure and Redox Imbalance

Mitochondrial failure and redox imbalance serve as downstream hubs where Aβ and tau toxicities converge to execute cell death. Microglial NOX2 activation and direct intracellular mitochondrial poisoning by Aβ, which binds to amyloid‐β‐binding alcohol dehydrogenase (ABAD), inhibit ETC complex IV, causing bioenergetic collapse and a reciprocal surge in mitochondrial superoxide leakage (Du et al. 2008; Lustbader et al. 2004). This oxidative environment induces lipid peroxidation within neuronal membranes, generating 4‐hydroxy‐2‐nonenal (4‐HNE), which disables essential Na^+^/K^+^‐ATPase pumps and destabilizes synaptic potentials (Markesbery 1997). Furthermore, ROS accumulation oxidizes cysteine residues within tau, promoting template‐directed tau aggregation independent of upstream kinase activity (Schweers et al. 1995). Ultimately, prolonged opening of the mitochondrial permeability transition pore (mPTP) releases cytochrome c, executing caspase‐3‐dependent apoptosis in vulnerable pyramidal neurons (Du et al. 2008) (Table 1).

**TABLE 1:** Overview of neurodegenerative diseases and their contributing factors.

Disease	Affected regions	NI	OS	Protein aggregation	Genetic factors	References
Alzheimer's disease (AD)	Hippocampus, prefrontal cortex, temporal lobe	Microglial activation; elevated IL‐1β, TNF‐α	Increased ROS, mitochondrial dysfunction, and reduced antioxidant enzymes	Amyloid‐β (Aβ) plaques, hyperphosphorylated tau tangles	APOE ε4, *PSEN1/2*, *APP*	Rajendran and Krishnan (2024), Twarowski and Herbet (2023), L. G. Yang et al. (2023), Das (2025)
Parkinson's disease (PD)	Substantia nigra, basal ganglia	Astrogliosis, TNF‐α, and IL‐6 elevation	Mitochondrial complex I inhibition, glutathione depletion	α‐Synuclein (Lewy bodies)	*SNCA*, *LRRK2*, *PARK2*	Day and Mullin (2021), Vrijsen et al. (2023), Kalyanaraman et al. (2024), Brooker and Gonzalez‐Latapi (2025)
Amyotrophic lateral sclerosis (ALS)	Motor neurons in the motor cortex and the spinal cord	Activated microglia, increased IL‐6, and TNF‐α	Elevated ROS, impaired glutamate metabolism	TDP‐43, SOD1 aggregates	*SOD1*, *C9orf72*, *TARDBP*	Goutman et al. (2022), Feldman et al. (2022), Akçimen et al. (2023), Ilieva et al. (2023), Rizea et al. (2024)
Multiple sclerosis (MS)	White matter of the brain and spinal cord	T‐cell and B‐cell activation, macrophage infiltration	Elevated nitric oxide and superoxide levels	Not covered	*HLA‐DRB1*, *IL2RA*, *IL7R*	McGinley et al. (2021), J. H. Yang et al. (2022), Haki et al. (2024), Prapas and Anagnostouli (2024), Garton et al. (2024)
Huntington's disease (HD)	Striatum, cerebral cortex	Chronic microglial activation	Mitochondrial dysfunction (complexes II/III), ROS production	Mutant huntingtin (mHTT) inclusions	*HTT* (expanded CAG repeats)	Stoker et al. (2022), A. Jiang et al. (2023), Tong et al. (2024), Mashayekhi and Salehi (2025)

Together, these interconnected events (Aβ‐driven synaptic toxicity, kinase‐mediated tau hyperphosphorylation, mitochondrial failure, and neuroinflammatory amplification) constitute a self‐reinforcing pathogenic cascade that progressively executes neuronal death in AD, serving as the archetypal model of convergent neurodegeneration discussed throughout this review.

### 3.2 Parkinson's Disease: Dysregulation and Neuronal Vulnerability

#### 3.2.1 Disease‐Specific Initiating Events and Pathogenic Cascade

PD has emerged as the fastest‐growing neurological disorder globally, with the current caseload exceeding 10 million individuals. Elucidating the precise mechanisms underlying this rapid epidemiological expansion remains an urgent biomedical priority. PD is defined by the selective loss of dopaminergic neurons within the substantia nigra pars compacta (SNpc) and the intracellular accumulation of α‐synuclein into Lewy bodies (Dong‐Chen et al. 2023; Spillantini et al. 1997). Pathogenesis is driven by the conformational shift of monomeric α‐synuclein, encoded by the *SNCA* gene, into β‐sheet‐rich soluble oligomers and higher‐order fibrils, a process accelerated by phosphorylation at Serine 129 (Fujiwara et al. 2002; Polymeropoulos et al. 1997)

In dopaminergic neurons, this aggregation cascade is uniquely exacerbated by dopamine metabolism byproducts, including reactive dopamine quinones and endogenous salsolinol (H. Wang et al. 2025; Wu et al. 2025). Salsolinol binds to the hydrophobic core of α‐synuclein, physically stabilizing toxic oligomeric intermediates and preventing their isolation into larger inclusions (Naoi and Maruyama 2022; Shayanfard et al. 2024).

These stabilized oligomers insert into synaptic vesicle membranes, causing vesicle leakage and a dying‐back axonal degeneration at striatal terminals (Minakaki et al. 2020; Volles and Lansbury 2003).

#### 3.2.2 Integration With Shared Neuroinflammatory Signaling

Extracellular α‐synuclein oligomers function as DAMPs that engage microglial TLR2 and TLR8, triggering the MyD88‐dependent activation of the IKK complex and subsequent nuclear translocation of NF‐κB (Kim et al. 2013; Lee et al. 2012). This pathway drives the transcriptional upregulation of TNF‐α, IL‐1β, and iNOS (Dutta et al. 2021; Hirsch and Hunot 2009). Concurrently, internalized α‐synuclein disrupts endo‐lysosomal membranes, activating the cytosolic NLRP3 inflammasome and caspase‐1 (Codolo et al. 2013). Sustained microglial secretion of TNF‐α and nitric oxide (NO) polarizes neighboring nigral astrocytes into the neurotoxic A1 phenotype, which exhibits an upregulation of NOX4 and pumps reactive species into the extracellular space (Liddelow et al. 2017). This chronic neuroinflammation represses neuroprotective pathways, including the BDNF/TrkB cascade, compromising dopaminergic survival (Hirsch and Hunot 2009).

#### 3.2.3 Integration With Mitochondrial Failure and Redox Imbalance

The unique vulnerability of SNpc dopaminergic neurons is tied to a circuit involving complex I failure and iron‐driven oxidative destruction. Intracellular α‐synuclein oligomers translocate to the inner mitochondrial membrane and directly inhibit ETC complex I, causing immediate ATP depletion and superoxide generation (Devi et al. 2008; Schapira et al. 1990). This is exacerbated by mutations in mitochondrial quality control genes, such as *PINK1* and *PRKN*, which prevent the mitophagic clearance of these dysfunctional organelles (Kitada et al. 1998; Valente et al. 2004). Compounding this, the SNpc contains elevated concentrations of labile ferrous iron (Fe^2+^) (Dexter et al. 1989). When complex I failure elevates cellular hydrogen peroxide, excess iron drives the Fenton reaction, yielding highly destructive hydroxyl radicals (OH) (Halliwell 1992). The resulting oxidative stress causes widespread lipid peroxidation, while carbonylation disables the 26S proteasome, further accelerating α‐synuclein aggregation and terminating in ferroptosis (Minakaki et al. 2020).

Crucially, the intersection of dopamine metabolism and proteostatic failure introduces a tissue‐specific vulnerability in dopaminergic neurons. Endogenous dopamine auto‐oxidation generates reactive dopamine quinones (DAQs) and neurotoxic derivatives such as salsolinol. Rather than merely acting as bystander molecules, DAQs specifically interact with the hydrophobic core of monomeric α‐synuclein, predominantly targeting the non‐amyloid‐β component (NAC) domain. This covalent modification or non‐covalent binding prevents the structural transition of α‐synuclein from disordered monomers into organized, insoluble β‐sheet fibrils. Consequently, DAQs kinetically trap and stabilize highly cytotoxic, soluble α‐synuclein oligomers, disrupting membrane integrity and accelerating mitochondrial and synaptic dysfunction before large‐scale Lewy body deposition occurs (Conway et al. 2001; Galkin et al. 2023).

### 3.3 ALS: The Frontiers of Cellular Stress and Failure

#### 3.3.1 Disease‐Specific Initiating Events and Pathogenic Cascade

ALS is a rapidly progressive and uniformly fatal motor neuron disease with a global incidence of approximately 1.5–2.5 per 100,000 person‐years. Despite being classified as a rare disease, its aggressive mortality rate and the severe clinical decline of patients make the elucidation of its shared mechanisms a high biomedical priority. The primary etiology revolves around proteostatic failure driven by heterogeneous mutations in *TARDBP*, *FUS*, *SOD1*, and *C9orf72* hexanucleotide repeat expansions (Neumann et al. 2006; Renton et al. 2011; Rosen et al. 1993). Neuropathologically, over 95% of cases exhibit cytoplasmic aggregation and nuclear clearance of TAR DNA‐binding protein 43 (TDP‐43) (Neumann et al. 2006). Pathological TDP‐43 undergoes hyperphosphorylation and cleavage into C‐terminal fragments, driving its nuclear‐to‐cytoplasmic translocation, where it partitions into liquid‐liquid phase‐separated stress granules (Verde et al. 2025). Chronic cellular stress transitions these dynamic assemblies into irreversible amyloid aggregates, inducing nuclear depletion, causing pre‐mRNA splicing defects (e.g., loss of stathmin‐2) and a toxic gain of function (GOF) where aggregates obstruct nuclear pores, halting protein translation (Polymenidou et al. 2011; Woerner et al. 2016).

#### 3.3.2 Integration With Shared Neuroinflammatory Signaling

**FIGURE 3:**
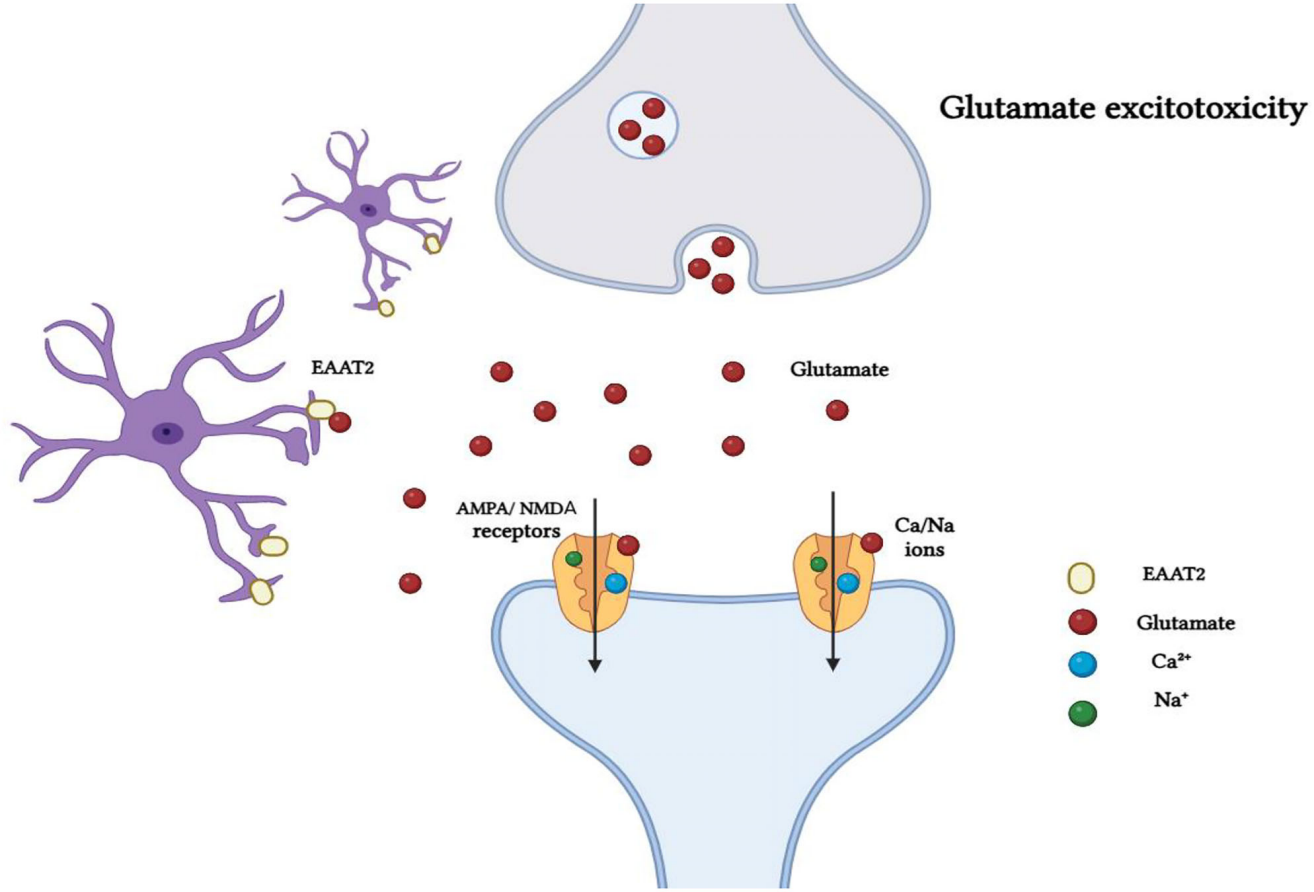
Mechanism of glutamate excitotoxicity in neurons. Pathological elevation of extracellular glutamate overstimulates postsynaptic AMPA and NMDA receptors, leading to excessive influx of Ca^2^^+^ and Na^+^ ions and subsequent neuronal injury or death. Astrocytic EAAT2 transporters are responsible for glutamate clearance from the synaptic cleft, thereby protecting against excitotoxic damage.

ALS progression is heavily driven by non‐cell autonomous toxicity arising from glial–neuronal crosstalk (Majewski et al. 2025). Motor neurons under proteostatic strain release misfolded proteins and DAMPs that engage microglial TLR4 and RAGE, inducing an M1 pro‐inflammatory state and NLRP3 inflammasome activation (Boillée et al. 2006; Gao et al. 2023). Concurrently, surrounding reactive spinal astrocytes undergo transcriptional silencing and degradation of the EAAT2/GLT‐1 transporter, preventing proper synaptic glutamate clearance (Rothstein et al. 1995). The resulting extracellular accumulation of glutamate triggers excitotoxicity via overactivation of neuronal AMPA and NMDA receptors (Van Damme et al. 2003). Because motor neurons express low levels of the Ca^2+^ permeability‐modulating GluR2 subunit, this astrocytic defect induces an uncontrolled intracellular Ca^2+^ influx, activating destructive calpains and caspases that dismantle the neurofilament network and initiate axonal degeneration (Lobsiger and Cleveland 2007; Van Damme et al. 2003) (Figure 3).

#### 3.3.3 Integration With Mitochondrial Failure and Redox Imbalance

Motor neurons have extraordinary axonal lengths and volumes, rendering them sensitive to bioenergetic collapse and oxidative stress (Le Gall et al. 2020). In ALS, mutant proteins (such as mutant SOD1 or mislocalized TDP‐43) associate with the outer mitochondrial membrane, disrupting the physical links at mitochondria‐associated membranes (MAMs) and compromising calcium homeostasis (Stoica et al. 2014; Vande Velde et al. 2008). These mutant proteins inhibit ETC complexes II and IV, halting oxidative phosphorylation, draining ATP reserves, and driving mitochondrial ROS accumulation (Vande Velde et al. 2008). Damaged mitochondria subsequently open the mPTP, releasing oxidized mtDNA and cardiolipin into the cytoplasm (Rousseau et al. 2025). Cytosolic mtDNA engages the cGAS‐STING pathway, which functions as a molecular bridge that amplifies iNOS transcription and fuels microglial inflammatory signaling (Yu et al. 2020). This ongoing oxidative injury carbonylated structural neurofilaments, arresting axonal transport and terminating in cell death (Le Gall et al. 2020) (Table 1).

### 3.4 Multiple Sclerosis: Immune System Misdirection and Consequences

#### 3.4.1 Disease‐Specific Initiating Events and Pathogenic Cascade

MS is the leading cause of nontraumatic neurological disability in young adults, affecting approximately 2.9 million people globally. While historically considered purely demyelinating, its progressive neurodegenerative phase forms the core of its long‐term disability, necessitating a comprehensive integration with shared neurodegenerative pathways. The initial cascade is triggered by a complex interplay of genetic vulnerabilities (primarily the HLA‐DRB1*15:01 haplotype) and environmental factors like EBV infection and vitamin D deficiency (Bjornevik et al. 2022; Menegatti et al. 2021). This matrix breaks peripheral tolerance, driving the expansion of autoreactive myelin‐specific CD4^+^ (Th1/Th17) and CD8^+^ T cells (Boutitah‐Benyaich et al. 2025). These lymphocytes upregulate α4β1 integrin (VLA‐4) to cross the BBB (Yednock et al. 1992). Once inside the CNS, reactivation by local antigen‐presenting cells presenting myelin epitopes (e.g., MBP, PLP) triggers a destructive immune response: Th1 cells secrete IFN‐γ, Th17 cells release IL‐17, and CD8+ T cells deploy perforin and granzyme B, inducing targeted cytolysis of oligodendrocytes and stripping axons of their insulation (Kirschner et al. 2025; Trapp et al. 1998).

#### 3.4.2 Integration With Shared Neuroinflammatory Signaling

As MS advances into its chronic progressive phase, inflammation shifts from acute peripheral infiltration to a compartmentalized, self‐sustaining process behind a repaired BBB (Cairns et al. 2022). Resident microglia, chronically activated by myelin breakdown products such as galactocerebroside, organize into inflammatory cuffs around damaged axons and continuously secrete high concentrations of TNF‐α and IL‐1β (Cairns et al. 2022; Frischer et al. 2009). This microenvironment induces a reactive transformation in surrounding astrocytes, polarizing them into the neurotoxic A1 phenotype (Liddelow et al. 2017). A key consequence of this astrocytic polarization is the profound transcriptional silencing of the EAAT2 glutamate transporter, severely undermining glutamate homeostasis within the inflamed matter (Pajarillo et al. 2021). The resulting accumulation of extracellular glutamate triggers widespread excitotoxicity; elevated glutamate overactivates axonal AMPA and NMDA receptors, provoking a destructive intracellular calcium surge that activates calpains and directly mediates axonal transection (Pitt et al. 2000; Trapp et al. 1998). Beyond acute structural damage, this persistent ionic destabilization and inflammatory environment eventually trigger a profound collapse of proteostatic machinery within demyelinated axons. The unremitting influx of calcium abnormally activates proteolytic enzymes, degrading critical neurofilaments and generating misfolded protein substrates that easily overwhelm the cellular clearance systems.

#### 3.4.3 Integration With Mitochondrial Failure and Redox Imbalance

Demyelinated axons face severe metabolic exhaustion and redox imbalance (Askari et al. 2024). Once demyelinated, axons must structurally redistribute voltage‐gated sodium channels along their entire length to maintain action potential propagation, creating a massive continuous demand for ATP (Askari et al. 2024). However, this demand cannot be met because microglial‐derived pro‐inflammatory cytokines and nitric oxide directly damage mitochondrial complexes I and IV, forcing a metabolic crisis (Silva et al. 2024). This deficit is accompanied by significant ROS generation. Oligodendrocytes are vulnerable to this redox imbalance due to low expression of antioxidant enzymes (CAT, GPx) and high intrinsic iron stores required for myelination. Exposed to ROS, this iron catalyzes Fenton chemistry, generating toxic hydroxyl radicals that induce DNA damage and lipid peroxidation, halting remyelination, a process worsened by a systematic failure of the Nrf2 antioxidant defense pathway (Margoni et al. 2022; Thorburne and Juurlink 1996). This oxidative microenvironment profoundly impairs local protein quality control mechanisms (Phan et al. 2023). Sustained elevation of ROS and RNS levels induces extensive carbonylation and nitration of axonal proteins, thereby compromising the ubiquitin‐proteasome system (UPS) and disrupting autophagic flux (Nakamura et al. 2021). The resultant accumulation of misfolded and aggregated proteins within oligodendrocytes and damaged axons ultimately converges on a common pathway of cell death (Lei and Lin 2024). This shared proteostatic failure establishes a mechanistic parallel between MS and classical proteinopathies, including AD and PD (Table 1).

### 3.5 Huntington's Disease: Genetic Defect to Molecular Dysfunction

#### 3.5.1 Disease‐Specific Initiating Events and Pathogenic Cascade

HD is a less prevalent autosomal dominant disorder, affecting approximately 1 in 10,000 individuals in Western populations. Due to its complete penetrance, HD serves as an ideal monogenic model for investigating the temporal dynamics of proteostatic failure and subsequent neurodegeneration. The monogenic cause of HD is an unstable CAG trinucleotide repeat expansion (> 36) within exon 1 of the *HTT* gene, which translates into an expanded polyglutamine (polyQ) tract within the N‐terminus of the huntingtin (HTT) protein (The HD Collaborative Research Group 1993). Under physiological conditions, wild‐type HTT acts as a scaffold coordinating vesicle trafficking and promoting the axonal transport of BDNF to striatal targets. The polyQ expansion induces a toxic GOF; the mutant huntingtin (mHTT) undergoes proteolytic cleavage by caspases and calpains, liberating highly toxic N‐terminal fragments (Andrew et al. 1993; Stoker et al. 2022; Tong et al. 2024). These fragments escape proteasomal clearance, assembling into soluble β‐sheet oligomers and inclusion bodies that sequester essential transcription factors (CBP and TBP), causing transcriptional silencing and the selective degeneration of GABAergic medium spiny neurons (MSNs) (DiFiglia et al. 1997; Scherzinger et al. 1997).

#### 3.5.2 Integration With Shared Neuroinflammatory Signaling

The proteostatic collapse induced by mHTT triggers chronic neuroinflammation that accelerates striatal atrophy. mHTT expression within resident microglia intrinsically lowers their activation threshold (Crotti et al. 2014). When exposed to mHTT aggregates released from degenerating MSNs, these primed microglia exhibit increased activity of the IKK/NF‐κB pathway, driving the continuous expression and secretion of pro‐inflammatory cytokines (IL‐1β, IL‐6, TNF‐α) (Wilton et al. 2023). Concurrently, internalized mHTT aggregates engage the microglial NLRP3 inflammasome, sustaining caspase‐1 activation (Ding et al. 2025). The resulting inflammatory microenvironment polarizes surrounding striatal astrocytes into the neurotoxic A1 phenotype. These reactive astrocytes exhibit a significant reduction in the expression of the potassium channel K and the glutamate transporter EAAT2/GLT‐1, preventing proper clearance and inducing severe excitotoxicity via overactivation of MSN NMDA receptors, triggering a massive calcium influx that completes the loss of MSNs (Fão et al. 2022; Pajarillo et al. 2021).

#### 3.5.3 Integration With Mitochondrial Failure and Redox Imbalance

The structural toxicity of mHTT converges on mitochondrial integrity and redox defenses, driving metabolic failure in HD (López Couselo et al. 2025). Soluble N‐terminal mHTT fragments physically bind to the outer mitochondrial membrane of MSNs, inhibiting respiratory complexes II and III (López‐Molina et al. 2025). This inhibition causes bioenergetic failure, characterized by a significant drop in ATP synthesis and elevated leakage of mitochondrial superoxide radicals (Panov et al. 2002). MSNs are vulnerable to this crisis due to high metabolic requirements and dependency on cortical BDNF support, which is reduced in HD (López Couselo et al. 2025). The resulting accumulation of ROS inflicts severe oxidative damage, heavily compounded by an intrinsic impairment of the Nrf2‐mediated antioxidant pathway (Chan et al. 2023). mHTT binds to and inactivates PGC‐1α, a master regulator of mitochondrial biogenesis, preventing the transcriptional activation of downstream Nrf2 target genes like *SOD2* and *GPX1*, leaving HD neurons defenseless against ongoing oxidative stress and completing the degeneration of striatal circuits (Chan et al. 2023) (Table 1).

## 4 Therapeutic Implications

Although oxidative stress and neuroinflammation are common across most NDDs, their relative contribution and initiating events differ from one disorder to another. This overlap, however, provides a strong rationale for developing multi‐target, disease‐modifying therapies rather than relying solely on symptomatic treatments.

Disease‐specific approaches have shown only partial and temporary benefits. In AD, anti‐Aβ monoclonal antibodies like donanemab reduce amyloid plaques and modestly slow cognitive decline. Yet their effectiveness is limited when treatment starts after significant neuronal loss has already occurred. They also have little effect on tau pathology, oxidative stress, or chronic neuroinflammation (Espay et al. 2024; van Dyck et al. 2023). In PD, iron chelators such as deferiprone and anti‐α‐synuclein strategies can reduce oxidative damage and protein aggregation, but they frequently fail to interrupt the broader vicious cycle involving mitochondrial dysfunction, glial activation, and sustained inflammation (B. Liu et al. 2024; Price et al. 2023). In ALS, approved drugs like riluzole and edaravone only modestly slow functional decline. They do not sufficiently address proteostatic failure, mitochondrial impairment, or harmful glial neuronal interactions (Pioro et al. 2024; Tzeplaeff et al. 2023). In MS, dimethyl fumarate provides clear benefits in relapsing‐remitting forms by activating Nrf2 and suppressing NF‐κB, but it does not fully halt progressive axonal degeneration caused by mitochondrial energy failure (Okuda et al. 2023). In HD, gene‐lowering therapies such as AMT‐130 are promising, yet their long‐term impact will likely be greater when combined with treatments targeting oxidative stress and inflammation (Mwape et al. 2026).

These limitations point to a clear need for multi‐target strategies that exploit the shared mechanisms discussed throughout this review. Because AD, PD, ALS, MS, and HD all involve chronic neuroinflammation, redox imbalance, mitochondrial dysfunction, impaired proteostasis, and excitotoxicity, therapies that modulate several of these nodes simultaneously are more likely to succeed. Activation of the Nrf2‐ARE pathway is particularly promising. This approach strengthens antioxidant defenses, reduces oxidative damage to proteins and lipids, decreases pro‐inflammatory cytokine production, and improves mitochondrial function. It has already proven clinically useful in MS and shows potential for broader application across other NDDs when used in combination regimens (Rauchová 2021; T. Wang et al. 2024). However, chronic or nonselective Nrf2 activation carries a risk of suppressing apoptosis in preneoplastic cells, underscoring the need for targeted delivery strategies. Pharmacological inhibition of the NLRP3 inflammasome is another attractive convergent target, given its central role in amplifying IL‐1β and IL‐18 signaling, driving microglial activation, and sustaining the feed‐forward loop between inflammation and oxidative stress in PD, AD, HD, and ALS (Amo‐Aparicio et al. 2023). However, broad NLRP3 suppression may impair beneficial innate immune responses, particularly in the context of infection or injury, highlighting the need for disease stage‐specific inhibition strategies. In addition, mitochondria‐targeted antioxidants (such as MitoQ or coenzyme Q10 derivatives) and agents that enhance autophagy can help clear toxic protein aggregates, including Aβ, tau, α‐synuclein, TDP‐43, and mutant huntingtin. Combining these modalities (for instance, Nrf2 activators with NLRP3 inhibitors and mitochondrial protectors) may more effectively disrupt the self‐amplifying pathogenic networks that drive progressive neuronal loss. Such combination therapies are valuable because they address both disease‐specific triggers and the common downstream mechanisms. Nevertheless, the major translational barrier remains the lack of validated biomarker panels capable of guiding patient selection and monitoring multi‐target treatment effects simultaneously.

Several important challenges must be overcome before these strategies reach the clinic. First, determining the optimal timing of treatment remains difficult. Neuroinflammatory and oxidative processes often begin many years before symptoms appear, yet most clinical trials enroll patients at later stages (Cummings et al. 2022; Jack et al. 2018). Second, marked patient heterogeneity (arising from different genetic backgrounds (e.g., *APOE4* in AD, *LRRK2* or *GBA* in PD, *C9orf72* in ALS), disease subtypes, and comorbidities) has led to inconsistent trial results. This highlights the urgent need for biomarker‐driven patient stratification (Hampel et al. 2023). Third, practical obstacles, including poor BBB penetration, off‐target effects (such as amyloid‐related imaging abnormalities with anti‐Aβ antibodies), potential drug–drug interactions in combination therapies, and safety concerns, continue to impede progress (van Dyck et al. 2023). It is also worth noting that nonspecific anti‐inflammatory drugs like NSAIDs have largely failed in large‐scale trials. They do not target disease‐specific mechanisms and may suppress beneficial aspects of the immune response. These failures further emphasize the need for precision medicine approaches.

Looking forward, the future of therapeutic development in NDDs lies in precision, mechanism‐based combination strategies that transcend traditional disease boundaries. By simultaneously modulating Nrf2‐driven antioxidant responses, NLRP3‐mediated inflammation, mitochondrial integrity, and proteostatic clearance (while carefully optimizing intervention timing and employing biomarker‐driven patient stratification), it may finally become possible to achieve meaningful disease modification. Success will also depend on innovative trial designs, such as adaptive platform trials, together with reliable biomarkers (e.g., neurofilament light chain levels, PET imaging of microglial activation, and CSF markers of oxidative stress) to monitor treatment effects (Hampel et al. 2023; Q. Jiang et al. 2025).

## 5 Conclusion

This review highlights a striking commonality across NDDs, including AD, PD, ALS, MS, and HD. Despite their distinct clinical presentations and initiating molecular mechanisms, these conditions share a deeply interconnected pathological framework. Key processes such as glial activation, oxidative stress, bioenergetic dysfunction, protein homeostasis disruption, and excitotoxic signaling are not isolated events but form self‐reinforcing feedback loops. This interconnectedness helps explain why neuronal degeneration continues unabated even after the primary causative factor has been triggered. Each disease‐specific analysis within this review reaffirms the central idea that regardless of whether the initial insult involves amyloid misprocessing, iron overload, RNA‐binding protein mislocalization, autoimmune attacks on myelin, or polyglutamine expansion, the downstream pathways driving neurodegeneration rely on overlapping molecular mechanisms.

From a therapeutic standpoint, this convergence of pathological mechanisms reshapes the approach to treatment. Targeting single molecules in a specific disease has historically produced only modest, short‐lived outcomes, reflecting the robust resilience of the underlying network rather than a failure of individual therapeutic targets. A more effective approach might integrate disease‐specific treatments with tools that address shared amplification nodes—mechanisms such as Nrf2‐mediated antioxidant enhancement, NLRP3‐driven inflammatory cascades, or mitochondrial bioenergetic defects. To translate these mechanistic insights into viable clinical outcomes, three strategic paradigms must be established. First, therapeutic intervention must be initiated during the preclinical phases when neural plasticity is still preserved. Second, patient populations must be stratified based on specific cellular and molecular endophenotypes rather than generic clinical diagnoses. Third, a robust panel of biomarkers must be identified to monitor network‐wide pathogenic alterations, not just one protein. Progress in these three areas will be essential for converting the mechanistic insights presented in this review into clinical strategies that genuinely alter disease trajectories, rather than only managing their symptoms.

## Author Contributions

**Fatemeh sadat Aldaghi**: conceptualization, drawing figure, writing – original draft, writing – review and editing, supervision. **Zakieh Siahpoosh**: review and editing, supervision. **Zivar Salehi**: conceptualization, writing – review and editing. **Farhad Mashayekhi**: review and editing, supervision. **Shabnam Sohrabnezhad**: review and editing, supervision. All authors contributed to the study conception and design.

## Funding

The authors have nothing to report.

## Ethics Statement

The authors have nothing to report.

## Conflicts of Interest

The authors declare no conflicts of interest.

## Data Availability

Data sharing is not applicable to this article, as no new data were generated or analyzed in this review.
